# Telerobotic Versus Standard Ultrasound in the Assessment of the Abdomen and Pelvis: A Real-World Prospective Study

**DOI:** 10.1155/ijta/1482326

**Published:** 2024-12-14

**Authors:** David Luengo Gómez, Ángela Salmerón Ruiz, María Isabel Romero Manjón, Antonio Medina Benítez, Antonio Jesús Láinez Ramos-Bossini

**Affiliations:** ^1^Abdominal Radiology Unit, Department of Radiology, Hospital Universitario Virgen de las Nieves 18014, Granada, Spain; ^2^Advanced Medical Imaging Group (TeCe-22), Instituto de Investigación Biosanitaria de Granada (ibs.GRANADA) 18012, Granada, Spain; ^3^Department of Human Anatomy and Embryology, School of Medicine, University of Granada 18071, Granada, Spain

**Keywords:** abdomen, telemedicine, teleradiology, telerobotics, ultrasound

## Abstract

**Introduction:** Telerobotic ultrasound has emerged as a promising technology in medicine, especially in settings with limited medical access or a lack of specialized personnel. However, there are very few studies evaluating its usefulness in real-world clinical practice.

**Objective:** This study evaluates the usefulness of abdominopelvic telerobotic ultrasound in a real-world practice setting.

**Methods:** A prospective study was performed in a cohort of adult patients who underwent abdominal ultrasound in a remote secondary hospital for suspected abdominal or pelvic pathology. Examinations were performed by an on-site technician and a remote abdominal radiologist. Satisfaction of patients and explorers, scan times, quality of visualization of anatomical structures, and ultrasound findings were measured and compared with standard ultrasound examinations performed by an on-site radiologist blinded to telerobotic ultrasound findings. Multivariate analyses were performed to predict variables related to the visualization quality of abdominopelvic organs.

**Results:** The sample included 40 patients (60% women; mean age, 51.2 ± 16.1 years; 35% overweight and 17.5% obese). Significant differences in ultrasound duration were observed between telerobotic ultrasound and standard ultrasound (27.4 ± 8.3 and 12.7 ± 3.1 min, respectively; *p* < 0.001). The mean satisfaction of radiologists, technicians, and patients with telerobotic ultrasound was high (7.35 ± 1.14 for radiologists, 7.93 ± 0.83 for technicians, and 8.43 ± 1.38 for patients). Visualization of anatomical structures was acceptable for most organs on telerobotic ultrasound but significantly worse than conventional ultrasound when “excellent visualization” was the reference standard. In addition, telerobotic ultrasound did not identify potentially relevant findings in a significant (70%) proportion of patients.

**Conclusions:** Telerobotic ultrasound offers acceptable results in the assessment of abdominopelvic organs and can help provide adequate healthcare to patients in locations with limited access to radiology specialists. However, there are significant limitations compared to standard ultrasound for their optimal evaluation.

## 1. Introduction

Ultrasound is one of the main imaging techniques used in medicine and is characterized by the advantages of being noninvasive, low-cost, and free of ionizing radiation, which makes this test an undoubtedly useful tool in clinical practice [1]. However, it suffers from some limitations, including the need for substantial experience and training and interobserver variability [2].

Remote ultrasound guided by telerobotic articulated systems (telerobotic ultrasound) is a technology in continuous development for more than two decades that makes it possible to perform ultrasound scans remotely using a device capable of reproducing the movement of the sonographer's arm [3]. This technology can have a remarkable impact in settings where healthcare is limited, such as in rural areas [4], offshore oil platforms, military stations, or aircraft [5]. Moreover, it has played a relevant role during the COVID-19 pandemic, as it has allowed the performance of ultrasound scans on patients in isolation [6].

The use of these systems has shown efficacy and cost-effectiveness in different anatomical regions and indications, including thyroid, carotid, prenatal, echocardiography, and abdominal ultrasound [7]. However, the introduction of this technology into routine clinical practice may be limited by several disadvantages, including a longer scanning time [8] or worse visualization of structures compared to standard ultrasound [9]. Some studies have reported comparable results for these potential limitations using remote abdominal ultrasonography, but the need for further studies has been emphasized [10].

There is a growing limitation in the availability of radiology specialists dedicated to abdominal ultrasound in second-level hospitals and peripheral health centers due to factors such as the lack of radiologists, the greater interest in specialization in third-level hospitals, or the international variability in the qualification requirements for professionals dedicated to ultrasound (sonographers, radiologists, obstetricians, cardiologists, etc.) [11]. All this has led to a growing interest in assessing the usefulness of telerobotic ultrasound in the context of nonurgent care demand.

From a resource management point of view, demonstrating the utility and cost-effectiveness of telerobotic ultrasound could be a paradigm shift by enabling appropriate waiting list logistics in a centralized manner. There is currently a range of commercial telerobotic ultrasound scanners that can be implemented in clinical practice [12–14]. However, the studies available to date are limited and do not yet offer the necessary guarantees to patients in terms of diagnostic quality, scan times, and doctor–patient communication under the radiological quality criteria typical of our environment.

The aim of this work is to evaluate the usefulness of remote ultrasound of the abdomen using an articulated telerobotic system in a real clinical practice setting through a prospective study.

## 2. Methodology

### 2.1. Patient Selection

This prospective study was approved by the Provincial Ethics Committee of Granada (Code 1235-N-20). A consecutive cohort of patients attended at the Hospital de Alta Resolucion de Alcala la Real (secondary hospital), located 58.9 km from the Hospital Universitario Virgen de las Nieves (reference hospital), who were requested to undergo an ultrasound examination of the abdomen and pelvis by the primary care physician between March 1 and April 1, 2022, were selected. Patients were invited to participate in the study on a voluntary basis. The inclusion criteria were as follows:

‐ Age > 18 years old.
‐ Suspected abdominal or pelvic pathology.
‐ Locality located >50 km from a tertiary-level hospital.

The exclusion criteria were as follows:

‐ Suspected musculoskeletal pathology.
‐ Request for ultrasound on a preferential or urgent basis.
‐ Patient's refusal to participate in the study.

### 2.2. Image Acquisition

The MELODY System (AdEchoTech) telerobotic system was used, which consists of an articulated arm installed in the ultrasound room, next to the table where the patient is positioned [10]. The articulated arm makes it possible to reproduce the remote movements of the hand reliably (360° of precession, 45° of nutation, and 360° of self-rotation) with an accuracy equal to or less than 1° [15].

The ultrasound equipment used was a SonixTablet (BK Ultrasound) equipped with a high-frequency linear probe (5–13 MHz) and a low-frequency convex probe (2–6 MHz). All the scans in the study were performed with the convex probe with a standardized abdominal imaging protocol.

**Figure 1:**
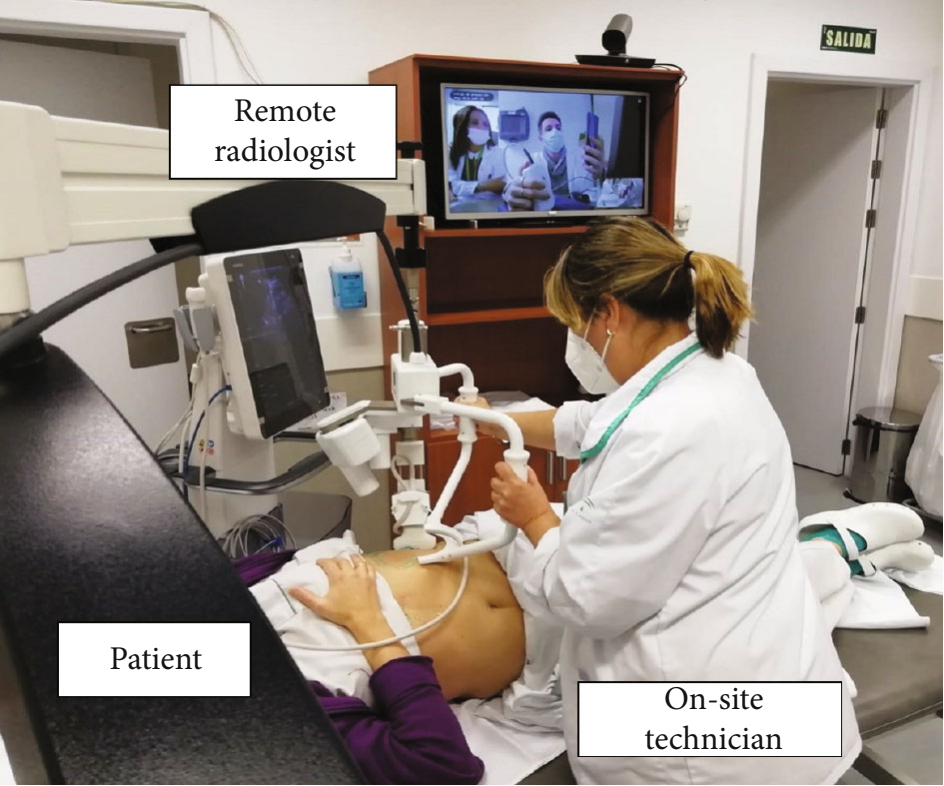
Telerobotic system used in the study (Melody, AdEchoTech). Technician placing the ultrasound probe with the patient in the supine position and on-screen remote radiologists performing telerobotic ultrasound.

Each ultrasound was performed by an on-site radiology technician and an abdominal radiologist at the referral hospital. The ultrasound protocol followed in the study is described in File S1. Using a high-speed Internet connection system and two touch screens, the technician, the radiologist, and the patient communicated via videoconference during the examination (Figure 1). After a training period of 2 weeks with 30 volunteer patients (not included in the study), two radiologists with experience in abdominal ultrasound (5 and 10 years) and two radiology technicians performed scheduled ultrasound shifts over a 4-week period. Patients underwent standard ultrasound immediately after the telerobotic ultrasound examination by an on-site radiologist with 10 years of experience who was blinded to the telerobotic ultrasound findings.

### 2.3. Study Variables

The following patient demographic variables were collected: age, sex, and BMI. For each scan, the indication, the quality of visualization of the main organs (*excellent*, *good*, *acceptable*, *poor*, and *lousy*), the imaging findings for each organ, and the scan time were collected. Finally, the radiologist, technician, and patient were asked to evaluate their degree of satisfaction with the test on a Likert scale from 0 to 10 points (lowest and highest possible degree of satisfaction). The meaning of the scores applied by the radiologist is shown in Table 1.

In the case of the technician survey, the following items were asked to be scored from 0 (lowest agreement) to 10 (highest agreement):

• “The use of the telerobotic tele-echography equipment has been useful to me as a healthcare professional.”
• “It has been easy to get familiar with using the equipment.”
• “It has been easy to use the equipment.”
• “Communication with the remote healthcare partner has been adequate for me.”

In the case of the patient, the following items were asked to be scored from 0 (lowest agreement) to 10 (highest agreement):

• “The examination has been comfortable.”
• “I would repeat the examination in the future.”

Subsequently, the same variables were evaluated in the standard ultrasound, except for satisfaction. Finally, the findings not visualized in either of the two ultrasound scans were collected for each organ examined.

### 2.4. Statistical Analysis

A descriptive study of the patient sample was performed. Qualitative variables were expressed as absolute and relative frequencies and quantitative variables as mean and standard deviation. A bivariate analysis was then performed comparing the variables measured in telerobotic and regulated ultrasound. Qualitative variables were analyzed by McNemar's test and quantitative variables by Student's *t*-test for related samples. In addition, a correlation analysis was performed on the quantitative measurements of both kidneys and the spleen using Pearson's *R* coefficient.

Finally, multivariate analyses were performed for the prediction of the variables “no visualization of a potentially relevant finding,” “not excellent visualization of an anatomical structure,” and “radiologist recommends regulated ultrasound” (the latter was obtained by dichotomizing the radiologist's satisfaction using values < 8 and ≥ 8 as cut-off points). A significance value of *p* < 0.05 was established. All analyses were performed with the SPSS Version 23.0 statistical package.

## 3. Results

The final sample consisted of a total of 40 patients (60% women) with a mean age of 51.2 ± 16.1 years. The mean BMI was 25.89, being normal in 19 patients (47.5%), overweight in 14 patients (35%), and obese in seven patients (17.5%). The clinical indications for ultrasound examination were suspected cholelithiasis (12 patients), renal lithiasis (18 patients), liver steatosis (six patients), and benign prostatic hyperplasia (four patients). The mean examination time in telerobotic ultrasound was 27.4 ± 8.3 min, and in standard ultrasound, it was 12.7 ± 3.1 min (significant differences; *p* < 0.001).

The mean satisfaction with telerobotic ultrasound was 7.35 ± 1.14 for radiologists, 7.93 ± 0.83 for technicians, and 8.43 ± 1.38 for patients (data not shown).

**Figure 2:**
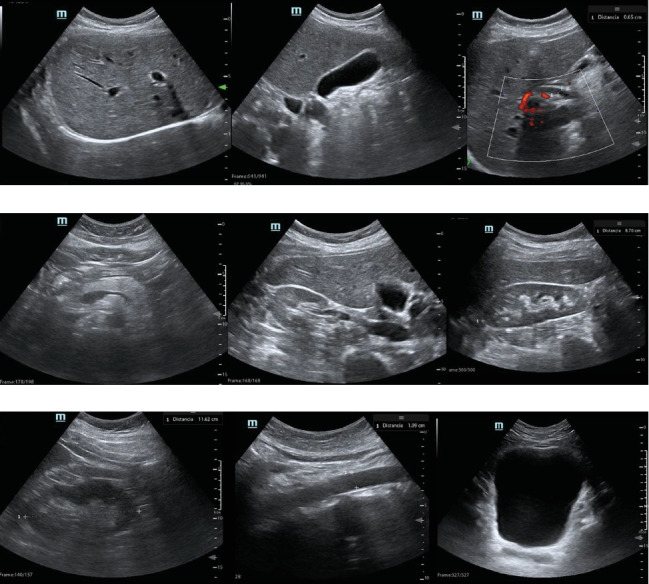
Examples of anatomical structures visualized on telerobotic ultrasound. (a) (left to right) liver, gallbladder, and main bile duct. (b) Pancreas and right kidney (axial and longitudinal views). (c) left kidney, aorta, and urinary bladder.

**Figure 3:**
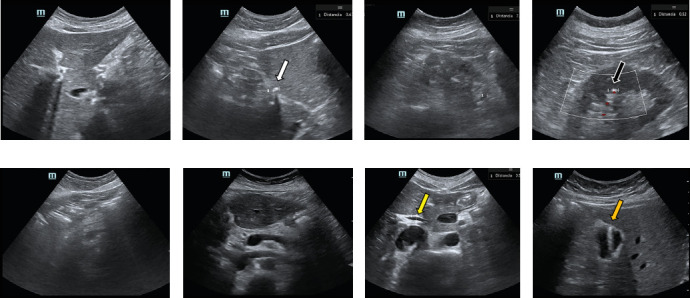
Illustrative examples of images obtained with telerobotic and standard ultrasound in our study. (a, b) Axial images at the level of the caudate and right lobe of the liver with (a) telerobotic and (b) standard ultrasound in a 44-year-old female patient with suspected choledocholithiasis. Despite the image, the quality of the images is similar (rated as “*good*” and “*excellent*” by the operators, respectively), and the 6-mm granuloma shown in (b) (white arrow) was not identified on telerobotic ultrasound. (c, d) Longitudinal images of the left kidney obtained with (a) telerobotic and (d) standard ultrasound in a 56 male patient with suspected renal lithiasis. The former image is of poor quality (rated as “*intermediate*” by the operator) and precluded the identification of the lithiasis shown in (b) (black arrow). (e, f) Axial images at the level of the pancreas on (e) telerobotic and (f) standard ultrasound. The pancreatic body shown in (f) could not be assessed on telerobotic ultrasound, which only enabled proper visualization of the (e) pancreatic head. (g) Example of bile duct (yellow arrow) measurement in standard ultrasound that could not be assessed on telerobotic ultrasound. (h) Focal adenomyomatosis of the gallbladder fundus (orange arrow) identified in telerobotic ultrasound that was missed on standard ultrasound.

The visualization of the different anatomical structures on telerobotic and standard ultrasound is shown in Table 2. Almost all organs were visualized with good or excellent quality in the latter case, with some exceptions in the pancreas (*n* = 4, 10%), biliary tract (*n* = 1, 2.5%), and uterus (*n* = 1, 4.8%). Illustrative examples of anatomical structures with good or excellent visualization on telerobotic ultrasound are provided in Figure 2. Illustrative examples of visualization of abdominal organs and findings on both ultrasound modalities are shown in Figure 3.

Table 3 shows the comparison of the visualization categories expressed as means of the overall score assigned per organ between both examinations. The mean overall score for visualization was significantly higher using standard ultrasound in each organ except for the gallbladder, with overall significant differences between groups (*p* < 0.001).

Table 4 shows the comparison between the “*excellent*” and “*good* or *excellent*” visualization categories between the two groups. For “*excellent*” visualization of organs, there were significant differences in all organs. However, for the combined category (good or excellent), significant differences were observed favoring standard ultrasound only for the biliary tract (*p* < 0.001), pancreas (*p* = 0.008), and uterus (*p* = 0.031).

Table 5 shows the findings identified in telerobotic ultrasound that were not observed in standard ultrasound and vice versa, grouped by organs. The total number of patients with findings identified on telerobotic ultrasound that were not detected on standard ultrasound was nine (22.5%), while the total number of patients with findings identified on standard ultrasound that were not detected on telerobotic ultrasound was 28 (70%). The corresponding figures for potentially relevant findings were one (2.5%) and 16 (40%).

The correlation analysis for the quantitative measurements (length) of the kidneys and spleen in both ultrasound modalities showed a statistically significant correlation between both ultrasound modalities (left kidney, *p* < 0.001; right kidney, *p* = 0.009; spleen, *p* < 0.01) (File S2).

The results of the multivariate analysis for the dependent variable “absence of identification of a potentially relevant finding in telerobotic ultrasound,” adjusted for age, sex, BMI, and scan time, were not statistically significant (File S3).

Table 6 shows the results of the multivariate analysis using logistic regression for the variable “nonexcellent visualization of anatomical structures.” Telerobotic ultrasound was a risk factor for not excellent visualization in all organs, while increased BMI was a risk factor in the visualization of the liver (adjusted odds ratio (ORa) 1.23; 95% confidence interval (CI), 1.02–1.48), gallbladder (ORa, 1.63; 95% CI, 1.25–2.13), and spleen (ORa, 1.22; 95% CI, 1.00–1.49).

Finally, the results of the multivariate analysis for the need to perform a standard abdominal ultrasound after telerobotic ultrasound, adjusted for age, sex, BMI, and scan time, were not statistically significant (File S4).

## 4. Discussion

The feasibility of telerobotic ultrasonography has been demonstrated in the evaluation of obstetric ultrasonography [16, 17], with some studies reporting favorable results in thyroid [18], carotid [19], and cardiopulmonary [20] assessment. Its usefulness has also been evaluated specifically in abdominal ultrasonography, but with some limitations that undermine the external validity of the results obtained [3, 10, 21]. In addition, to our knowledge, in previous studies, ultrasound examinations were performed by sonographers but not by abdominal radiology specialists.

Our results show that, despite the longer time required for each ultrasound scan and the lower sensitivity for the overall detection of findings, telerobotic ultrasound can be useful for the assessment of abdominopelvic pathologies in different organs with acceptable results, in line with previous studies [3, 10, 19, 21]. Notably, although the radiologists followed a standardized protocol for the assessment of all abdominal organs, we found that the visualization quality of telerobotic ultrasound varies depending on the organ being assessed, with similar results to standard ultrasound in the gallbladder, aorta, urinary bladder, and spleen and slightly inferior—although acceptable—outcomes in the liver and kidneys. The latter organs were of notable importance in our sample, taking into account that more than 60% of the clinical indications for ultrasound were related to suspected renal lithiasis or hepatic steatosis.

The greatest limitations were found in the assessment of the biliary tract, pancreas, and uterus. These findings are slightly different from those reported by Adams et al., who found that the organs that were worst visualized on telerobotic ultrasound were the pancreas, aorta, spleen, gallbladder, and left kidney [10]. Surprisingly, in the said study, the bile duct and liver were visualized in a comparable manner between the two examinations. This could be explained by methodological differences in the design and patient sample between both studies. Overall, we hypothesize that the poorer visualization of abdominal organs in telerobotic ultrasound was mainly due to intrinsic operator–dependent factors (i.e., the need to synchronize the technician with the radiologist's instructions) and equipment-related factors (limited pressure application, which cannot be sensed by the radiologist, and inability to perform translational displacements on the patient's surface without the technician).

The mean duration of telerobotic ultrasound examinations was similar to that described in some previous studies [10], although other studies have reported shorter scanning times [19]. One study by Ren et al. in China using a telerobotic ultrasound system with 5G technology reported a mean duration of 7–11 min and concluded that the telerobotic system employed was useful and feasible for rural population health screenings [22]. Scan times shorter than ours (i.e., 12.2 ± 4.5 min) were also reported in the study by Chai et al. with similar equipment and compared to “bedside” ultrasound. These authors reported that this system showed similar cost-effectiveness [21]. Based on our results and experience, such scan times should only be acceptable for specific assessments of target organs (e.g., liver and biliary tract, kidneys, and urinary bladder). The longer duration of telerobotic ultrasound in our study could be attributed to factors such as inefficient coordination between the radiologist and the technician, the intrinsic limitations of the robotic arm mentioned above, or most likely, the extensive effort to achieve excellent visualization of the organs, given that the ultrasound standards of the abdominal radiologist are thorough and of high quality. Of note, despite longer duration times, satisfaction rates in our sample were high for patients and explorers.

Interestingly, our multivariate analysis to predict the variable “nonexcellent visualization of anatomical structures” showed that telerobotic ultrasonography was a risk factor for all organs and high BMI was a risk factor in the visualization of liver, gallbladder, and spleen. In our opinion, the importance of BMI has not been explored with sufficient attention in previous studies on telerobotic ultrasound, and it is an important factor to consider, particularly in countries with high obesity rates, as it has been shown to worsen the visualization of different organs and lesions [23]. In fact, one of the most important technical limitations of telerobotic ultrasound derives from the inability to control the degree of pressure and transducer displacement [3, 10]. These limitations may be particularly important in the case of overweight or obese patients, where it is necessary to exert a greater degree of pressure and to use several viewing windows.

Overall, our results highlight the need to ensure the professional competence of radiologists in remote secondary hospitals when implementing telerobotic ultrasound. In our study, all remote ultrasound examinations were performed by abdominal radiologists, which ensured high standards of image interpretation and diagnostic accuracy. However, in routine practice, ensuring consistent professional quality in remote settings may require different strategies, such as specific training and continuous education—including novel educational approaches for resource-limited settings [24]—or the development of specific telemedicine protocols similar to the telestroke guidelines [25]. These measures would help mitigate any potential variability in diagnostic performance due to geographic location or resource availability. Of note, the advantages of ultrasound compared to other imaging modalities such as CT or MRI (e.g., lack of ionizing radiation, low cost, and ability to detect specific conditions such as noncalcified cholelithiasis [26, 27]) enhance the need for improving efforts to preserve the use of this imaging modality in resource-limited setting such as second-level hospitals.

The main limitations of the study include the relatively low sample size, which probably affected the multivariate analyses, and the use of ad hoc questionnaires and scores. In addition, we did not address potential confounders such as patient comorbidities or operator experience. Of note, all radiologists involved in the study had significant expertise and similar experience in abdominal ultrasound. The main strengths are its prospective nature, the blinding, the application of the same ultrasound equipment, and the inclusion of abdominal radiologists as ultrasound performers.

Future prospective studies with larger numbers of patients are needed, including a broader representation of patients' physical characteristics (e.g., BMI) and clinical indications. Particularly important is the evaluation of the usefulness of telerobotic ultrasound in urgent abdominopelvic pathologies, since it could solve the need for on-site radiologists in rural areas and second-level hospitals distant from referral hospitals. In addition, the cost-efficiency of the equipment needs to be evaluated in a regulated manner. Although some studies have found telerobotic ultrasound to be cost-efficient in rural and remote areas where access to specialized medical care is limited [4], considering the current radiological quality standards, further studies should be performed to demonstrate the economical benefits of this technology in nonrural areas.

## 5. Conclusions

Telerobotic ultrasound allows an acceptable assessment of most abdominopelvic organs, but there are significant limitations compared to standard ultrasound for their optimal evaluation, especially in the pancreas, biliary tract, and uterus. Although this ultrasound modality can help provide adequate healthcare to patients in locations with limited access to radiology specialists, its longer duration and limited visualization of organs should be overcome to justify its widespread use in other medical environments such as second-level hospitals.

## Figures and Tables

**Table 1:** Interpretation of the satisfaction scale applied to radiologists who performed telerobotic ultrasound.

**Score**	**Meaning**
0	Examination not performed
1	No relevant abdominal anatomical structures can be adequately visualized
2	Only one organ can be adequately visualized
3	Only two or three organs can be adequately visualized
4	Only four or five organs can be adequately visualized
5	Only six or seven organs can be adequately visualized
6	Eight or nine organs can be adequately visualized
7	All organs can be visualized, but it is not possible to optimally assess any potentially relevant region given the clinical suspicion
8^a^	All organs can be visualized, but it is not possible to optimally assess some regions of no potential interest given the suspicion
9^a^	All organs can be optimally visualized and evaluated, except for some regions due to the patient's morphotype
10^a^	All organs can be optimally visualized and assessed

^a^In these cases, the sonographer would not recommend performing a regulated ultrasound.

**Table 2:** Visualization of anatomical structures in standard and telerobotic ultrasound.

**Organ**	**Visualization**				
	***Very poor***	***Poor***	***Acceptable***	***Good***	***Excellent***
	**N** ** (%)**	**N** ** (%)**	**N** ** (%)**	**N** ** (%)**	**N** ** (%)**
Standard ultrasound					
Liver	0 (0)	0 (0)	5 (12.5)	20 (50)	15 (37.5)
Gallbladder	0 (0)	0 (0)	0 (0)	18 (47.4)^a^	20 (52.6)^a^
Biliary tract	0 (0)	1 (2.5)	12 (30)	22 (55)	5 (12.5)
Right kidney	0 (0)	1 (2.5)	3 (7.5)	20 (50)	16 (40)
Left kidney	1 (2.5)	1 (2.5)	4 (10)	21 (52.5)	13 (32.5)
Pancreas	2 (5)	2 (5)	8 (20)	23 (57.5)	5 (12.5)
Spleen	0 (0)	0 (0)	0 (0)	12 (30)	28 (70)
Aorta	0 (0)	0 (0)	3 (7.5)	17 (42.5)	20 (50)
Urinary bladder	0 (0)	0 (0)	2 (5)	16 (40)	22 (55)
Prostate	0 (0)	0 (0)	3 (7.5)^a^	9 (22.5)^a^	4 (10)^a^
Uterus	0 (0)	1 (2.5)^a^	6 (15)^a^	9 (22.5)^a^	5 (12.5)^a^
Telerobotic ultrasound					
Liver	0 (0)	0 (0)	0 (0)	9 (22.5)	31 (77.5)
Gallbladder	0 (0)	0 (0)	0 (0)	1 (2.6)^a^	37 (97.4)^a^
Biliary tract	0 (0)	0 (0)	1 (2.5)	13 (32.5)	26 (65)
Right kidney	0 (0)	0 (0)	0 (0)	6 (15)	34 (85)
Left kidney	0 (0)	0 (0)	0 (0)	7 (17.5)	33 (82.5)
Pancreas	0 (0)	0 (0)	4 (10)	14 (35)	22 (55)
Spleen	0 (0)	0 (0)	0 (0)	3 (7.5)	37 (92.5)
Aorta	0 (0)	0 (0)	0 (0)	3 (7.5)	37 (92.5)
Urinary bladder	0 (0)	0 (0)	0 (0)	2 (5)	38 (95)
Prostate	0 (0)	0 (0)	0 (0)	2 (12.5)^a^	14 (87.5)^a^
Uterus	0 (0)	0 (0)	1 (4.8)^a^	5 (23.8)^a^	15 (37.5)^a^

^a^Percentage of total valid cases (excluding cases of cholecystectomy, hysterectomy, or sexual incompatibility in the case of reproductive organs).

**Table 3:** Comparison of visualization categories between telerobotic and standard ultrasound by anatomical structures.

**Organ**	**Telerobotic ultrasound**	**Standard ultrasound**	**p** ^∗^ ** value**
	**X** ** (SD)**	**X** ** (SD)**	
Liver	3.25 (0.67)	3.78 (0.42)	< 0.001
Gallbladder	3.53 (0.51)	3.63 (0.54)	0.254
Biliary tract	2.78 (0.70)	3.63 (0.54)	< 0.001
Right kidney	3.28 (0.72)	3.85 (0.36)	< 0.001
Left kidney	3.10 (0.87)	3.83 (0.39)	< 0.001
Pancreas	2.68 (0.94)	3.45 (0.68)	< 0.001
Spleen	3.70 (0.46)	3.93 (0.27)	0.002
Aorta	3.43 (0.64)	3.93 (0.27)	< 0.001
Urinary bladder	3.50 (0.60)	3.95 (0.22)	< 0.001
Prostate	3.06 (0.68)	3.88 (0.34)	< 0.001
Uterus	2.86 (0.85)	3.67 (0.58)	< 0.001
Overall	3.19 (0.69)	3.78 (0.42)	< 0.001

*Note:* Data are expressed as mean (*X*) and standard deviation (SD) out of 5 points, with 1 = *very poor visualization* and 5 = *excellent visualization* (see Table 2).

⁣^∗^*p* value for Student's *t*-test for paired samples.

**Table 4:** Comparison of good or excellent visualization between telerobotic and standard ultrasound by anatomical structures.

**Organ**	**Excellent visualization**			**Good or excellent visualization**		
	**Telerobotic ultrasound**	**Standard ultrasound**	**p** ** value**	**Telerobotic ultrasound**	**Standard ultrasound**	**p** ** value**
	**N** ** (%)**	**N** ** (%)**		**N** ** (%)**	**N** ** (%)**	
Liver	15 (37.5)	31 (77.5)	< 0.001	35 (87.5)	40 (100)	NS
Gallbladder	20 (52.6)^a^	37 (97.4)^a^	< 0.001	38 (100)^a^	38 (100)^a^	NS
Biliary tract	5 (12.5)	26 (65)	< 0.001	27 (67.5)	39 (97.5)	< 0.001
Right kidney	16 (40)	34 (85)	< 0.001	36 (90)	40 (100)	NS
Left kidney	13 (32.5)	32 (80)	< 0.001	34 (85)	40 (100)	NS
Pancreas	5 (12.5)	22 (55)	< 0.001	28 (70)	36 (90.0)	0.008
Spleen	28 (70)	37 (92.5)	0.004	40 (100)	40 (100)	NS
Aorta	20 (50)	37 (92.5)	< 0.001	37 (92.5)	40 (100)	NS
Urinary bladder	22 (55)	38 (95)	< 0.001	38 (93)	40 (100)	NS
Prostate	4 (10)^a^	14 (87.5)^a^	0.002	13 (81.3)	16 (100)	NS
Uterus	5 (12.5)^a^	15 (37.5)^a^	0.002	14 (66.7)	20 (95.2)	0.031

Abbreviation: NS, not significant.

^a^Percentage of total valid cases (excluding cases of cholecystectomy, hysterectomy, or sexual incompatibility in the case of reproductive organs). *p* value of McNemar's test.

**Table 5:** Findings observed in standard or telerobotic ultrasound that were not identified in the other modality.

**Findings by organs**	**Findings in telerobotic ultrasound not detected on standard ultrasound**	**Findings in standard ultrasound not detected on telerobotic ultrasound**
	**N** ** (%)**	**N** ** (%)**
Liver		
Simple cyst	1 (2.5)	**3 (7.5)**
Granuloma	2 (5)	**1 (2.5)**
Hemangioma	0	**1 (2.5)**
Others	—	—
Total	3 (7.5)	5 (12.5)
Gallbladder	0	0
Lithiasis	0	**1 (2.5)**
Biliary mud	0	**2 (5)**
Polyp	0	1^a^ (2.5)
Adenomyomatosis	1 (2.5)	0
Others	—	—
Total	1 (2.5)	4 (10)
Biliary tract	0	0
Ectasia	0	1 (2.5)
Choledocholithiasis	0	0
Others	—	—
Total	0	1 (2.5)
Right kidney	0	0
Simple cyst	2 (5)	4 (10)
Complex cyst	0	0
Nonobstructive lithiasis	0	**3 (7.5)**
Obstructive lithiasis	0	0
Others	—	—
Total	2 (5)	7 (17.5)
Left kidney	0	0
Simple cyst	2 (5)	4 (10)
Complex cyst	0	0
Nonobstructive lithiasis	**1 (2.5)**	**2 (5)**
Obstructive lithiasis	0	**1 (2.5)**
Others	—	—
Total	3 (7.5)	7 (17.5)
Pancreas	0	0
Cystic lesion	0	**1 (2.5)**
Wirsung duct ectasia	0	1 (2.5)
Others	—	—
Total	0	2 (5)
Spleen	0	0
Focal lesion	0	0
Mild splenomegaly	0	1 (2.5)
Others	0	0
Total	0	1 (2.5)
Aorta	0	0
Dilation	0	0
Atheromatosis	0	1 (2.5)
Others	0	0
Total	0	1 (2.5)
Urinary bladder	0	0
Outgrowth lesion	0	0
Wall bladder thickening	0	1 (2.5)
Others	0	0
Total	0	1 (2.5)
Uterus	0	0
Leiomyoma	0	0
Others	—	—
Total	0	0
Total number of patients with findings identified with respect to the other modality	9 (22.5)	28 (70)
Total number of patients with potentially relevant findings identified with respect to the other modality	1 (2.5)	16 (40)

*Note:* The findings in boldface are considered potentially relevant.

^a^Percentage with respect to the total number of patients.

**Table 6:** Multivariate analysis (logistic regression) for nonexcellent visualization of anatomical structures.

**DV: nonexcellent visualization**	**Telerobotic ultrasound (ref: standard ultrasound)**	**Age (years)**	**BMI (kg/m)** ^**2**^	**Male sex (ref: Female sex)**	**Scanning time (minutes)**
	**ORa (95% CI)**	**ORa (95% CI)**	**ORa (95% CI)**	**ORa (95% CI)**	**ORa (95% CI)**
Liver	12.13 (3.28–44.89)⁣^∗^	1.00 (0.96–1.05)	1.23 (1.02–1.48)⁣^∗^	4.89 (1.27–18.74)⁣^∗^	1.02 (0.95–1.10)
Gallbladder	29.00 (5.07–166.03)⁣^∗^	1.02 (0.96–1.07)	1.63 (1.25–2.13)⁣^∗^	0.49 (0.10–2.39)	1.00 (0.91–1.09)
Biliary tract	11.47 (3.68–35.66)⁣^∗^	1.02 (0.98–1.06)	1.08 (0.91–1.28)	1.23 (0.35–4.28)	0.99 (0.92–1.06)
Right kidney	9.39 (2.97–29.75)⁣^∗^	1.03 (0.99–1.07)	1.11 (0.95–1.31)	0.47 (0.13–1.65)	1.01 (0.95–1.08)
Left kidney	9.98 (3.30–30.16)⁣^∗^	0.96 (0.93–1.00)	1.11 (0.94–1.31)	0.62 (0.18–2.15)	1.01 (0.95–1.08)
Pancreas	8.69 (2.65–28.54)⁣^∗^	1.03 (0.99–1.08)	1.10 (0.92–1.31)	1.51 (0.42–5.41)	0.99 (0.92–1.06)
Spleen	5.94 (1.36–25.69)⁣^∗^	1.02 (0.98–1.07)	1.22 (1.00–1.49)⁣^∗^	0.75 (0.17–3.36)	0.92 (0.84–1.00)
Aorta	16.81 (3.77–74.90)⁣^∗^	1.04 (0.99–1.09)	1.15 (0.96–1.38)	1.35 (0.35–5.19)	0.95 (0.88–1.03)
Urinary bladder	18.29 (3.56–93.88)⁣^∗^	1.03 (0.99–1.08)	1.02 (0.86–1.23)	0.78 (0.19–3.12)	0.92 (0.85–1.00)
Prostate	72.32 (3.10–1685.75)⁣^∗^	1.10 (0.99–1.21)	1.03 (0.79–1.36)	—	0.86 (0.72–1.04)
Uterus	7.70 (1.73–34.26)⁣^∗^	1.01 (0.96–1.06)	1.25 (0.98–1.59)	—	1.04 (0.95–1.14)

Abbreviations: 95% CI, 95% confidence interval; BMI, body mass index; DV, dependent variable; ORa, adjusted odds ratio; Ref, reference group.

⁣^∗^Statistically significant values.

## Data Availability

All data are available upon reasonable request to the corresponding author.

## Supporting Information

Supporting Information Additional supporting information can be found online in the Supporting Information section. Supporting information include the ultrasound protocol followed and three tables containing data not shown in the main document that complement the study findings. File S1: Ultrasound protocol followed in the study. File S2: Correlation analysis of the longitudinal measurements of the kidneys and spleen in the two ultrasound modalities. Data in the first columns are expressed as mean (standard deviation). File S3: Results of the multivariate analysis for the nonidentification of potentially relevant findings using telerobotic ultrasound. ORa, adjusted odds ratio; 95% CI, 95% confidence interval; BMI, body mass index. File S4: Results of the multivariate analysis for the need to perform standard abdominal ultrasound after telerobotic ultrasound. ORa, adjusted odds ratio; 95% CI, 95% confidence interval; BMI, body mass index.
