# Supplementary material for: Telerobotic Versus Standard Ultrasound in the Assessment of the Abdomen and Pelvis: A Real-World Prospective Study
Source: Int J Telemed Appl. 2024 Dec 14;2024:1482326. doi: 10.1155/ijta/1482326 (PMC11663047; doi:10.1155/ijta/1482326)
Supplement: Supporting Information — Additional supporting information can be found online in the Supporting Information section. Supporting information include the ultrasound protocol followed and three tables containing data not shown in the main document that complement the study findings. File S1: Ultrasound protocol followed in the study. File S2: Correlation analysis of the longitudinal measurements of the kidneys and spleen in the two ultrasound modalities. Data in the first columns are expressed as mean (standard deviation). File S3: Results of the multivariate analysis for the nonidentification of potentially relevant findings using telerobotic ultrasound. ORa, adjusted odds ratio; 95% CI, 95% confidence interval; BMI, body mass index. File S4: Results of the multivariate analysis for the need to perform standard abdominal ultrasound after telerobotic ultrasound. ORa, adjusted odds ratio; 95% CI, 95% confidence interval; BMI, body mass index. [file 1482326.f1.docx]

| **Patient preparation**  Abdomen, pelvis and lower thorax clear, unclothed, for proper evaluation and approach.  It is recommended to fast for 6 hours, particularly for gallbladder evaluation. Adequate bladder filling is recommended (suggest fluid intake and not urinating 3 hours before ultrasound) to assess bladder and uterus or prostate via transabdominal approach.  **Equipment and technique**  Use of convex probes (frequency range: 2-6 MHz).  The patient will be placed in supine decubitus at the beginning of the examination. Throughout the examination, right and left lateral decubitus positions may be required with the arm above the head, and even complementary examinations in prone, standing or sitting position. Likewise, their collaboration will be requested with deep inspirations and apneas that allow better assessment of the different organs. If necessary, use maintained compression.  The equipment presets used are those provided by default for abdominal examination. These presets are suitable for standard patients. If necessary, gray scale, general gain, frequency and dynamic range can be modified according to the needs of the study and the patient's body constitution.  **Study registration**  All study findings will be documented with sufficient images or videos to allow its interpretation and comparisons in further studies. Minimum essential images shall include:   - Left hepatic lobe: axial and longitudinal images covering all its segments; assessment in longitudinal sections of retro hepatic vena cava and aorta. - Right hepatic lobe: axial and longitudinal images identifying its segments. - Confluence of hepatic veins in inferior vena cava, branches of right portal vein. - Hepatic hilum: subcostal and/or intercostal approach. Assessment of main portal vein (diameter from internal wall to internal wall). - Gallbladder: axial and longitudinal sections. Wall thickness. Explore in supine and left oblique position to identify decanted and hidden lithiasis in the infundibular region and assess its mobility. - Biliary tract: axial image of confluence, anterior to portal bifurcation and hepatic arteries. Longitudinal image. Assess as distal as possible. Measure its diameter (height of its crossing with the right hepatic artery) from internal wall to internal wall. - Pancreas: identify its different portions, head, body and tail. Identify and measure, if seen, the pancreatic duct. - Spleen: Longitudinal image with measurement. Optional axial images. - Kidneys: longitudinal images with measurements. Optional axial images. In case of lithiasis suspicion, color Doppler may be used to search for twinkling artifact (decrease color Doppler frequency and increase speed range). - Retroperitoneum: axial images with measurement of aortic diameter (measure from external wall to external wall). Longitudinal image of the aorta. - Pelvis: assessment of uterus (and ovaries, if seen) or prostate, and abdominal fluid in Douglas space.   Any findings described in the report (focal lesions, lithiasis, abdominal masses, collections, etc.) must be adequately documented and measured in different images. |
| --- |

**Supplementary File 1: Ultrasound protocol followed in the study**

| **Structure** | **Tele-robotic ultrasound**  **X (SD)** | **Standard ultrasound**  **X (SD)** | **Pearson's R** | **p-value** (Pearson's R correlation) | **p-value** (Student's t-test paired samples) |
| --- | --- | --- | --- | --- | --- |
| Left kidney | 11.25 (0.70) | 11.46 (0.56) | 0.896 | <0.001 | <0.001 |
| Right kidney | 10.47 (0.50) | 10.58 (0.67) | 0.931 | <0.001 | 0.009 |
| Spleen | 10.05 (1.33) | 10.26 (1.29) | 0.972 | <0.001 | <0.001 |

**Supplementary File 2.** Correlation analysis of the longitudinal measurements of the kidneys and spleen in the two ultrasound modalities**.** Data in the first columns are expressed as mean (standard deviation).

| **Variable** | **ORa (95%CI)** |
| --- | --- |
| Age | 1.02 (0.97-1.07) |
| BMI | 1.09 (0.89-1.33) |
| Sex (male) | 0.79 (0.17-3.57) |
| Ultrasound duration | 1.00 (0.92-1.09) |

**Supplementary File 3.** Results of the multivariate analysis for the non-identification of potentially relevant findings using tele-robotic ultrasound**.** ORa, adjusted odds ratio. 95% CI, 95% confidence interval. BMI, body mass index.

| **Variable** | **ORa (95%CI)** |
| --- | --- |
| Age | 0.98 (0.94-1.03) |
| BMI | 0.90 (0.74-1.10) |
| Sex (male) | 1.14 (0.26-5.04) |
| Ultrasound duration | 1.00 (0.92-1.09) |

**Supplementary File 4.** Results of the multivariate analysis for the need to perform standard abdominal ultrasound after tele-robotic ultrasound**.** ORa, adjusted odds ratio. 95% CI, 95% confidence interval. BMI, body mass index.
